# Supplementary material for: The Small RNA Universe of Capitella teleta
Source: Front Mol Biosci. 2022 Feb 25;9:802814. doi: 10.3389/fmolb.2022.802814 (PMC8915122; doi:10.3389/fmolb.2022.802814)
Supplement: Supplementary file 1 [file DataSheet1.ZIP › Supplement/known/CAPTEscaffold_85_6332.pdf]

Provisional ID	:	CAPTEscaffold_85_6332
Score total	:	21578.6
Score for star read(s)	:	3.9
Score for read counts	:	21569.3
Score for mfe	:	0.8
Score for randfold	:	1.6
Score for cons. seed	:	3
Total read count	:	42319
Mature read count	:	42218
Loop read count	:	0
Star read count	:	101

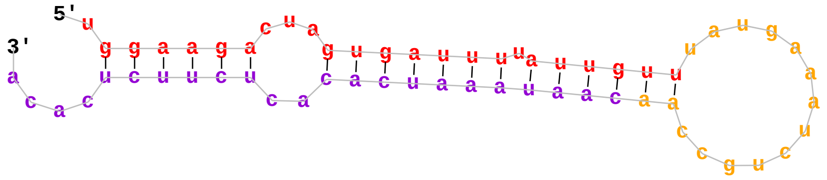

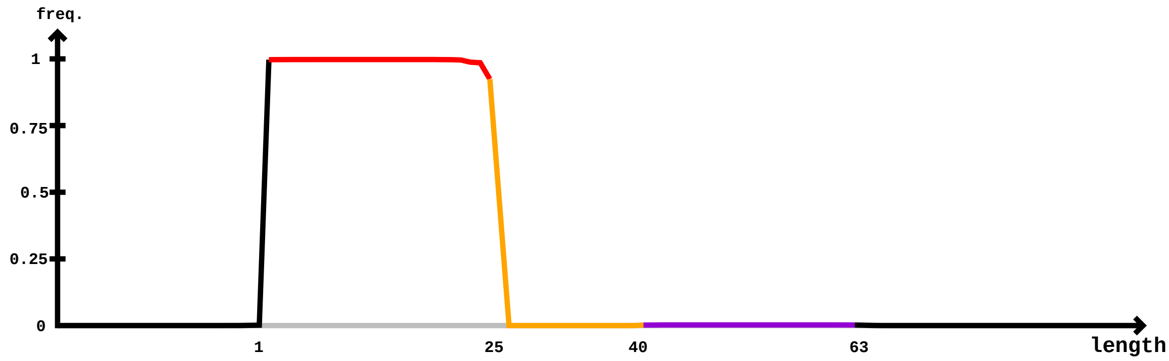

Mature Star

```
5'- uaacccucuguuucaugaugcuggaagacuagugauuuuauuguuuaugaaaucugccaacaauaaaucacacucuucucacaucauguacaguacuuacaacuuguauaau  -3'  obs
```

```
5'- uaacccucuguuucaugauguggaagacuagugauuuuauuguuuaugaaaucugccaacaauaaaucacacucuucucacaucauguacaguacuuacaacuuguauaau  -3'  obs
    uaacccucuguuucaugauguggaagacuagugauuuuauuguuuaugaaaucugccaacaauaaaucacacucuucucacaucauguacaguacuuacaacuuguauaau       exp
    uaacccucuguuucaugauguggaagacuagugauuuuauuguuuaugaaaucugccaacaauaaaucacacucuucucacaucauguacaguacuuacaacuuguauaau       known
    .......((((..((((((((((((((...(((((((.((((((.............)))))))))))))..))))).))))))))).)))).................  reads  mm  sample
    uaacccucuguuucaugaug...........................................................................................  2  0  seq
    ................gauCuggaagacuagugauuuuauuguu...................................................................  1  1  seq
    ..................uguggaagacuagugauuuua........................................................................  1  0  seq
    ..................uguggaagacuagugauuuuau.......................................................................  2  0  seq
    ..................uguggaagacuagugauuuuauu......................................................................  27  0  seq
    ..................Gguggaagacuagugauuuuauuguu...................................................................  2  1  seq
    ...................Cuggaagacuagugauuuuauu......................................................................  11  1  seq
    ...................Cuggaagacuagugauuuuauuguu...................................................................  14  1  seq
    ....................uggaagacuagugauuuu.........................................................................  14  0  seq
    ....................uggaagacuagugauuuua........................................................................  7  0  seq
    ....................uggaagacuagugauuuuau.......................................................................  51  0  seq
    ....................uAgaagacuagugauuuuau.......................................................................  1  1  seq
    ....................uggaagacuagugauuuuauu......................................................................  311  0  seq
    ....................uggaagacuagAgauuuuauu......................................................................  1  1  seq
    ....................uggaagGcuagugauuuuauu......................................................................  1  1  seq
    ....................uggaagacuagugauuuuauug.....................................................................  81  0  seq
    ....................uggaagacuagugauGuuauug.....................................................................  1  1  seq
    ....................uggaagacuagugauuuuaAugu....................................................................  1  1  seq
    ....................uggaagacuagugauuuuauAgu....................................................................  4  1  seq
    ....................ugUaagacuagugauuuuauugu....................................................................  2  1  seq
    ....................uggaaAacuagugauuuuauugu....................................................................  1  1  seq
    ....................Aggaagacuagugauuuuauugu....................................................................  12  1  seq
    ....................uggaagacuagugauuuuaGugu....................................................................  1  1  seq
    ....................uggaagacuagugauAuuauugu....................................................................  3  1  seq
    ....................uggGagacuagugauuuuauugu....................................................................  3  1  seq
    ....................uggaagacuUgugauuuuauugu....................................................................  1  1  seq
    ....................uggaagacuagugauuuGauugu....................................................................  1  1  seq
    ....................uggaagacuagugauuAuauugu....................................................................  1  1  seq
    ....................ugCaagacuagugauuuuauugu....................................................................  1  1  seq
    ....................uggaagacuagugauuuuauugG....................................................................  1  1  seq
    ....................uggaagacuaguAauuuuauugu....................................................................  3  1  seq
    ....................uAgaagacuagugauuuuauugu....................................................................  13  1  seq
```

Mature Star

```
uaacccucuguuucaugauguggaagacuagugauuuuauuguuuaugaaaucugccaacaauaaaucacacucuucucacaucauguacaguacuuacaacuuguauaau
....................uggaagacuagugaGuuuauugu.................................................................... 1 1 seq
....................uCgaagacuagugauuuuauugu.................................................................... 2 1 seq
....................Cggaagacuagugauuuuauugu.................................................................... 1 1 seq
....................uggaagacuagugauuuuGuugu.................................................................... 1 1 seq
....................uggaUgacuagugauuuuauugu.................................................................... 1 1 seq
....................Gggaagacuagugauuuuauugu.................................................................... 1 1 seq
....................uggaagacuagugauuuuauugu.................................................................... 2537 0 seq
....................uggaagacuagAgauuuuauugu.................................................................... 1 1 seq
....................Cggaagacuagugauuuuauuguu................................................................... 12 1 seq
....................uggaagacuaUugauuuuauuguu................................................................... 1 1 seq
....................uggaagacuagugauuuuauuguC................................................................... 7 1 seq
....................uggaagacuagugauuAuauuguu................................................................... 17 1 seq
....................uggaagaAuagugauuuuauuguu................................................................... 3 1 seq
....................Aggaagacuagugauuuuauuguu................................................................... 201 1 seq
....................uggaagacuagAgauuuuauuguu................................................................... 15 1 seq
....................uggaagacuagCgauuuuauuguu................................................................... 6 1 seq
....................uggaaAacuagugauuuuauuguu................................................................... 8 1 seq
....................Nggaagacuagugauuuuauuguu................................................................... 10 1 seq
....................uggaagacuaguUauuuuauuguu................................................................... 6 1 seq
....................uggaagacGagugauuuuauuguu................................................................... 2 1 seq
....................uggaagacuagGgauuuuauuguu................................................................... 2 1 seq
....................uggaagacuagugGuuuuauuguu................................................................... 6 1 seq
....................uggaagacuagugauuuuauugCu................................................................... 4 1 seq
....................uggaagacuagugauuuuauuguu................................................................... 37962 0 seq
....................uggaagacuagugauuuCauuguu................................................................... 16 1 seq
....................uggaagacuagugauuuuaCuguu................................................................... 3 1 seq
....................uggaagacCagugauuuuauuguu................................................................... 5 1 seq
....................uggaagacuagugauCuuauuguu................................................................... 9 1 seq
....................uggaaCacuagugauuuuauuguu................................................................... 1 1 seq
....................ugAaagacuagugauuuuauuguu................................................................... 15 1 seq
....................uggaagacuaguCauuuuauuguu................................................................... 1 1 seq
....................uggaagacuagugauuuuauuguG................................................................... 11 1 seq
....................uggaagacuaCugauuuuauuguu................................................................... 1 1 seq
....................uggaagacuagugauuuuauGguu................................................................... 5 1 seq
....................uggUagacuagugauuuuauuguu................................................................... 4 1 seq
....................uggGagacuagugauuuuauuguu................................................................... 10 1 seq
....................uCgaagacuagugauuuuauuguu................................................................... 9 1 seq
....................uggaagacuagugauuuuGuuguu................................................................... 2 1 seq
....................uggaagacuagugauuuGauuguu................................................................... 2 1 seq
....................ugUaagacuagugauuuuauuguu................................................................... 4 1 seq
....................uggaagacuagugauuuuauCguu................................................................... 7 1 seq
....................uggaagacuagugaAuuuauuguu................................................................... 5 1 seq
....................uggaagacuagugauuuuauAguu................................................................... 13 1 seq
....................uggaagacAagugauuuuauuguu................................................................... 6 1 seq
....................uAgaagacuagugauuuuauuguu................................................................... 247 1 seq
....................uggaagacuagugauuuuauugAu................................................................... 12 1 seq
....................uUgaagacuagugauuuuauuguu................................................................... 2 1 seq
....................uggaagacuagugauGuuauuguu................................................................... 3 1 seq
....................uggaagacuagugauuuuauuAuu................................................................... 4 1 seq
....................uggaagacuGgugauuuuauuguu................................................................... 1 1 seq
....................uggaagGcuagugauuuuauuguu................................................................... 4 1 seq
....................uggaagacuagugauuuuaGuguu................................................................... 4 1 seq
....................uggaagacuagugauuGuauuguu................................................................... 1 1 seq
....................uggaagaGuagugauuuuauuguu................................................................... 1 1 seq
....................uggaagacuagugauAuuauuguu................................................................... 21 1 seq
....................uggaagacuagugauuuuauuguA................................................................... 16 1 seq
....................uggaagacuagugauuuuauuUuu................................................................... 4 1 seq
....................uggaagacuaguAauuuuauuguu................................................................... 79 1 seq
....................uggaagacuagugauuuuaAuguu................................................................... 6 1 seq
....................uggaagUcuagugauuuuauuguu................................................................... 5 1 seq
....................ugCaagacuagugauuuuauuguu................................................................... 13 1 seq
....................uggaGgacuagugauuuuauuguu................................................................... 13 1 seq
....................uggaagacuagugauuuuauugGu................................................................... 3 1 seq
....................uggaagacuagugaCuuuauuguu................................................................... 1 1 seq
....................uggaagacuagugauuCuauuguu................................................................... 5 1 seq
....................uggaagacuagugauuuuUuuguu................................................................... 1 1 seq
....................Gggaagacuagugauuuuauuguu................................................................... 39 1 seq
....................uggaagacuagugauuuAauuguu................................................................... 2 1 seq
....................uggaagacuaAugauuuuauuguu................................................................... 1 1 seq
....................uNgaagacuagugauuuuauuguu................................................................... 1 1 seq
```

Mature **Star**

```
uaacccucuguuucaugaugugggaagacuagugauuuuauuguuuaugaaaucugccaacaauaaaucacacucuucucacaucauguacaguacuuacaacuuguauaau
```

```
uaacccucuguuucaugauguggaagacuagugauuuuauuguuuaugaaaucugccaacaauaaaucacacucuucucacaucauguacaguacuuacaacuuguauaau
....................uAgaagacuagugauuuuauuguuu..................................................................  3   1   seq
....................uggaagacuagugauuuuauuguuA.................................................................. 89   1   seq
....................uggaagacuagugauuuuauuguuu.................................................................. 98   0   seq
....................uggaagacuagugauuuuauuguuC..................................................................  1   1   seq
....................uggaagacuagugauuuuauuguuAa.................................................................  1   1   seq
....................uggaagacuagugauuuuauuguuua.................................................................  1   0   seq
....................uggaagacuagugauuuuauuguuuU.................................................................  2   1   seq
.....................ggaagacuagugauuuuauuguu...................................................................  6   0   seq
.....................Ugaagacuagugauuuuauuguu...................................................................  5   1   seq
......................gaagacuagugauuuuauugu....................................................................  3   0   seq
......................gaagacuagugauuuuauuguu...................................................................  2   0   seq
.......................aagacuagugauuuuauugu....................................................................  1   0   seq
.......................aagacuagugauuuuauuguu...................................................................  5   0   seq
........................agacuagugauuuuauuguu...................................................................  1   0   seq
..........................acuagugauuuuauuguu...................................................................  2   0   seq
...........................................................caauaaaucacacucuucuc................................  1   0   seq
...........................................................caauaaaucacacucuucuca...............................  3   0   seq
...........................................................caauaaaucacacucuucucac..............................  8   0   seq
...........................................................caauaaaucacacucuucucacU.............................  3   1   seq
...........................................................caauaaaucacacucuucucaca............................. 65   0   seq
............................................................aauaaaucacacucuucucac..............................  1   0   seq
............................................................aauaaaucacacucuucucaca.............................  3   0   seq
............................................................aauaaaucacacucuucucacauU...........................  1   1   seq
.............................................................auaaaucacacucuucucaca.............................  2   0   seq
.............................................................auaaaucacacucuucucacau............................  1   0   seq
.............................................................auaaaucacacucuucucacauc........................... 12   0   seq
.............................................................auaaaucacacucuucucacauU...........................  1   1   seq
```
